# Juxtaposition of Mesenchymal Stem Cells with Endothelial Progenitor Cells Promoted Angiogenic Potential Inside Alginate-Gelatin Microspheres

**DOI:** 10.34172/apb.2021.017

**Published:** 2020-11-07

**Authors:** Shirin Saberianpour, Reza Rahbarghazi, Mahdi Ahmadi, Mohammad Nouri, Morteza Heidarzadeh, Abbas Karimi, Souror Nemati

**Affiliations:** ^1^Department of Molecular Medicine, Faculty of Advanced Medical Sciences, Tabriz University of Medical Sciences, Tabriz, Iran.; ^2^Stem Cell Research Center, Tabriz University of Medical Sciences, Tabriz, Iran.; ^3^Department of Applied Cell Sciences, Faculty of Advanced Medical Sciences, Tabriz University of Medical Sciences, Tabriz, Iran.; ^4^Chemical Engineering Faculty, Sahand University of Technology, Tabriz, Iran.

**Keywords:** Endothelial progenitor cells, Mesenchymal stem cells, Alginate-gelatin microspheres, Angiogenesis

## Abstract

***Purpose:*** Here, we investigated the angiogenic potential of endothelial progenitor cells juxtaposed with mesenchymal stem cells (MSCs) inside alginate-gelatin microspheres with stromal derived factor-1α (SDF-1 α) for 7 days.

***Methods:*** Six encapsulated groups were allocated including endothelial progenitor cells (EPCs), EPCs/SDF-1α, MSCs, MSCs/SDF-1α, EPCs+MSCs and EPCs+MSCs/SDF-1α. Cells were encapsulated with a mixture of 1% alginate and 2% gelatin hydrogel. Cell survival was examined by MTT assay. Endothelial differentiation was determined by flow cytometry and ELISA. Tubulogenesis assay and Ac-Dil-LDL uptake were used to detect functional activity. Cell migration was analyzed by Transwell insert and gelatin zymography analyses. By using real-time polymerase chain reaction (PCR), we measured the transcription of *Akt* and *PK1*.

***Results:*** We found an increase in cell viability in MSCs/SDF-1α microspheres compared to EPCs group (*P* <0.05). EPC/MSCs co-culture contributed to the increase of CD133+ cells while we found high CD31 levels in MSCs group (*P* <0.05). Juxtaposition of EPC with MSCs increased tubulogenesis compared to SDF-1a-free condition (*P* <0.001). SDF-1α had the potential to increase in AC-LDL uptake in MSCs and EPCs/MSCs groups. Cells migration and MMP-9 activities increased after treatment with SDF-1α. SDF-1α upregulated *PK1* and *Akt* in encapsulated cells, especially in a co-culture system.

***Conclusion:*** Alginate-gelatin microspheres could alter the angiogenic potential of progenitor cells in the presence of SDF-1α

## Introduction

Up to date, numerous technologies and developments have been raised in the field of tissue engineering to increase in favor of cell bioactivity inside the body.^1^ Of these modalities, cell microencapsulation has been rapidly expanded to immobilize cells with semi permeable membranes in a three-dimensional space for effective cell delivery into the target sites.^2^

Alginate, an anionic natural biopolysaccahride, is extensively used as a backbone for scaffolds and microspheres. However, cell attachment to scaffolds with the basis of alginate must be improved by the addition of extracellular matrix. Due to the potential of gelatin in providing specific motifs and the decrease of the anionic net charge of alginate, gelatin is commonly used in the field of tissue engineering.^3^ The simultaneous induction and maintenance of angiogenesis and vascularization toward administrated cell mass enable a high rate of cellular bioactivity.^4^ In a previous work conducted by our group, we found that encapsulation of human umbilical vein endothelial cells with alginate-gelatin microspheres led to the formation of vascular units in the muscular tissues.^5^ So, appropriate policies must be exploited to induce angiogenesis at a very short time post-cell transplantation. Based on the previous experiments, it has been demonstrated that mesenchymal stem cells (MSCs) and endothelial progenitor cells (EPCs) possess the capacity to induce angiogenesis in a juxtacrine and paracrine manner.^6^ In this regard, Li et al successfully examined the synchronous culture of MSCs and EPCs for induction of angiogenesis and osteogenesis.^7^ Among the critical angiogenesis factors, vascular endothelial growth factor (VEGF) and stromal derived factor-1α (SDF-1 α) and CXCR-4 axis are important to induce cell migration toward injured sites.^8^ SDF-1α could attach to CXCR-4 receptor and activate downstream signaling pathways notably PI3K/Akt axis participates in cell motility and migration. Here, we investigated the angiogenic potential after simultaneous encapsulation of human MSCs and EPCs inside alginate-gelatin microspheres. We also examine the effect of factor SDF-1α on the stemness and migration potential of encapsulated cells.

## Materials and Methods

### Umbilical cord blood samples

To isolate human EPCs, the umbilical blood samples were collected in tubes containing 2500 IU heparin and immediately transferred to the lab. Prior to EPCs isolation, all volunteers were asked to complete the informed consent.

### Cell isolation and expansion

#### EPCs

For isolation of EPCs, an equal volume of samples was diluted with PBS solution and gently overlaid on Ficoll-Hypaque solution. Thereafter, samples were centrifuged at 400 g for 30 minutes and cells at the interface between the Ficoll-Hypaque solution and supernatant were collected and washed three times with PBS. Cells were plated in each well of 6-well plates (SPL) pre-coated with human fibronectin (dilution: 1 μg/mL; Cat no: C-43060; PromoCell). To induce the growth of human EPCs, freshly isolated mononuclear cells were cultured in Endothelial Growth Medium-2 (EGM-2; Cat no: C-22211; PromoCell) supplemented with 10% fetal bovine serum and Growth Medium 2 Supplement Pack (Cat no: C-39211; PromoCell) and the medium was replenished every 3-4 days. After 7 days, CD133^+^EPCs were enriched by using MACS technique. For this propose, cells were collected and blocked with 1% FBS for 20 min and incubated with CD133 microbeads (Cat no: Miltenyi Biotec) for 30 min according to the manufacturer’s recommendation. Thereafter, cells were passed through the LS columns (Cat no: Miltenyi Biotec), collected in tubes and used for different assays.

#### MSCs

MSCs cell line (Cat no: IBRC-C10892) were purchased from Iranian Biological Resource Center. To expand MSCs, cells were incubated with low glucose Dulbecco’s Modified Eagle Medium (DMEM/LG; Gibco) supplemented with 10% FBS and 1% Pen-Strep. Cells at passage three were subjected to further analyses.

### Cell encapsulation with alginate-gelatin microcapsules

The basis of encapsulation methods is dipping the alginate-based solution into a liquid containing 1% calcium chloride. In the current experiment, cells were classified into six distinct groups including EPCs, EPCs-SDF-1α, MSCs, MSCs-SDF-1α, MSCs+ EPCs, and MSCs-EPCs-SDF-1α. To encapsulate cells, 1×10^6^ cells were mixed with 1 mL of 1% alginate+ 2% gelatin and aspirated by a syringe connected to a 25G needle. Syringe nozzle was connected to the positive part of the high-voltage generator and other to 1% CaCl_2_ solution. The voltage was set to 7 kV by using voltage electrolysis device (FnM, Hu35p oc, high voltage power supply). 0.2 mL/min flow rate was used for the generation of microcapsules. In the groups containing MSCs + EPCs, cells were encapsulated at a ratio of 1:1. To combine SDF-1α with the mixture of alginate-gelatin, 10 ng/mL SDF-1α was added per ml of alginate-gelatin mixture. After a 7-day incubation at conventional environment at 37°C with 5% CO_2_ and 95% relative humidity, microspheres were decapsulated by using 0.01 M sodium citrate trihydrate (Cat no: 6132-04-3) until the release of cells. Thereafter, cells were collected, washed twice with PBS and subjected to different analyses.

### Scanning electron microscopy

By using SEM imaging, we visualized the microspheres morphology. For this propose, we fixed microspheres in 2.5% glutaraldehyde (Merck) and washed in PBS. Thereafter, the samples snap frozen in freeze-dryer (Operon Co. Ltd., Korea). Gold was used for coating the samples and then monitored by SEM (Model: MIRA3 FEG-SEM, Tescan).

### Cell survival assay by MTT

At respect time point, cells were released inside of alginate-gelatin microcapsules from different groups and viability examined by MTT assay. In brief, 1 × 10^4^ cells were transferred onto each well of 96-well plates (SPL). Thereafter, 100 μL medium containing 5 mg/ml culture media powder (Sigma) was added to each well and kept at 37°C for 4 hours. Then, the supernatant was discarded, replaced with 100 μL of dimethyl sulfoxide (Merck) solution and agitated gently for 10 minutes. The optical density of wells was measured by an ELSA reader at 570 nm. Cell survival rate was reported as % of the control.

### Analysis of VEGFR-2 and CD133 by flow cytometry 

To detect the angiogenic potential of encapsulation, we performed flow cytometry analysis to determine the percentage of cells expressing endothelial marker CD31. The cells were collected from different groups and washed twice with PBS. To permeabilize the cells, we used permeabilizing buffer (Bioscience) at 4°C for 20 min and cells were incubated with a panel of antibodies against human CD133 (FITC-conjugated, Miltenyi Biotec) and CD31 (PE-conjugated; eBioscience) at 4°C for 30 min according to the manufacturer’s recommendation. After twice washing with PBS, cells were fixed by pre-cold paraformaldehyde solution (4%) for 10 min and analyzed by BD FACSCalibur system and FlowJo software (version 7.6.1).

### In vitro tube formation

To evaluate the angiogenesis potential of cells inside alginate-gelatin microspheres, we performed an *in vitro* tubulogenesis assay. First, each well of 96-well plates was filled with 50 µL pre-chilled Matrigel (Corning) and allowed to solidify at 37°C. Then, 2 × 10^4^ cells from each group were re-suspended in 100 μL culture medium supplemented with 1% FBS and transferred into each well. Cells were cultured at 37°C with 5% CO_2_ under humidified atmosphere for 8-24 h. After completion of this period, we measured the average of the tube area (µm^2^) in all groups and compared with each other.

### Monitoring the expression of AKT1 and PK genes by real-time PCR

To examine the expression of AKT1 and PK genes in all groups, the microcapsules were decapsulated at respective time points. RNA was extracted by using RNA extraction kit (Cat no: YT9065; YTA Co., Iran). The quality of RNAs was evaluated by using the Thermo Scientific NanoDrop™ 1000 system. The cDNA was synthesized by cDNA synthetase kit (Bioneer). Specific primers against genes AKT1 and PK were designed by Oligo7 software (Molecular Biology Insights Inc.). Real-time polymerase chain reaction (PCR) was performed by SYBR Green and MIC system (BioMolecular Systems, Australia. The transcription of each gene was calculated by comparing with housekeeping gene GAPDH. In this study, the 2^-ΔΔCT^ method was used. The primer list was outlined in Table 1.

**Table 1:** Primer list

**Gene**	**Sequences**	**Tm (°C)**
*AKT1*	F:5'-GAGGATGTGGACCAACGTGA-3' R:5'-AAGGTGCGTTCGATGACAGT-3'	60
*PROK1*	F:5'-CTGTGAGCGGGATGTCCAG-3' R:5'-GGTGCTTGCGTTTCCTGAAG-3'	59
*GAPDH*	F:5'-CCTGCACCACCAACTGCTTA-3' R:5'-AGTGATGGCATGGACTGTGG-3'	60

### Evaluation of cell migration by Transwell inserts 

For this propose, 2 × 10^4^ cells in 200 μL medium were transferred onto inserts. In the bottom wells, 700 μL medium supplemented with SDF-1α (10 ng/mL) and 1% FBS was added. After 24 h, the number of cells in each well was counted in 10-high-power-fields.

### Investigating the functional activity of endothelial cells using AC-LDL-Dil

One of the methods measures vascular cells bioactivity is based on the dynamics of lipase activity in cells differentiating into the endothelial lineage. For this propose, decapsulated cells from different groups were cultured on 8-well culture slide (SPL) for 8 hours and incubated with 100 μL of a dye fluorescent Dil (20 µM; Molecular Probes) at 37°C for 40 min. Thereafter, the cells were washed three times with PBS and 1 µg/mL of DAPI solution (Sigma) added to each well. Finally, images were visualized by using a Fluorescence microscopy (Model: BX41; Olympus). Finally, the number of double positive red and blue cells was counted at 10 random high-power fields per group and compared with each other.

### Zymography

We also performed zymography assay to evaluate the activity of metalloproteinase-2 and -9 (MMP-2 and MMP-9) post-encapsulation with alginate-gelatin microspheres. At the respective time point, cells were lysed by using protein lysis buffer at 4°C overnight. The next days, samples were centrifuged at 14 000 g for 20 minutes and supernatants used for the analysis. For this end, 100 μg protein from each was electrophoresed on 12% SDS-PAGE gel containing 0.2% gelatin. Thereafter, gels were incubated twice with 0.5% Triton X100 (each for 30 min). To reactivate MMP-2 and MMP-9, we used zymography buffer containing 50 mM Tris-HCl, (pH 7.4), 5 mM CaCl2, and 0.02% NaN3. After 24 h, 0.2% Coomassie blue dye solution was used to stain the gels. To remove excess stain after use, gels were washed with a destaining solution (0.1% acetic acid). Finally, gels were scanned by an HP Scanjet G3110 apparatus (Hewlett-Packard Company). Scanned images were set to black and white in Adobe Photoshop software CS5 (Middle Eastern ver. 12.0 × 32) and the density of bands was measured by using ImageJ (NIH; version 1.4.). All experiments were performed in triplicate.

### Measuring endothelial differentiation of encapsulated cells based on VE-cadherin

To investigate the endothelial differentiation of encapsulated cells after 7 days being enclosed by the alginate-gelatin hydrogel, we measure the protein content of VE-cadherin by ELISA. In brief, 100 μL protein was transferred onto each well of polystyrene 96-well plates (SPL) and maintained at 4°C overnight. Next, wells were blocked with 1% FBS for 1 h. Thereafter, we added 100 μL mouse anti-human VE-cadherin antibody (dilution 1: 100; Abcam) and kept for 1 h. After twice washing with PBS, we added HRP conjugated anti-mouse IgG secondary antibody (1: 1000; Abcam), incubated for 30 min and washed three times with PBS. 3, 3’, 5, 5’-Tetramethylbenzidine was used as chromogenic substrate and the reaction stopped by using 5% H_2_SO_4._ Finally, the absorbance was read at 450 nm by using microplate readers (BioTek).

### Statistical analysis

Data are shown as mean ± SD. To find the statistical differences, we performed one-way ANOVA and Tukey post hoc analysis. In this study, *P*  < 0.05 was considered statistically significant.

## Results

### Cell morphology and microsphere diameter

**Figure 1:**
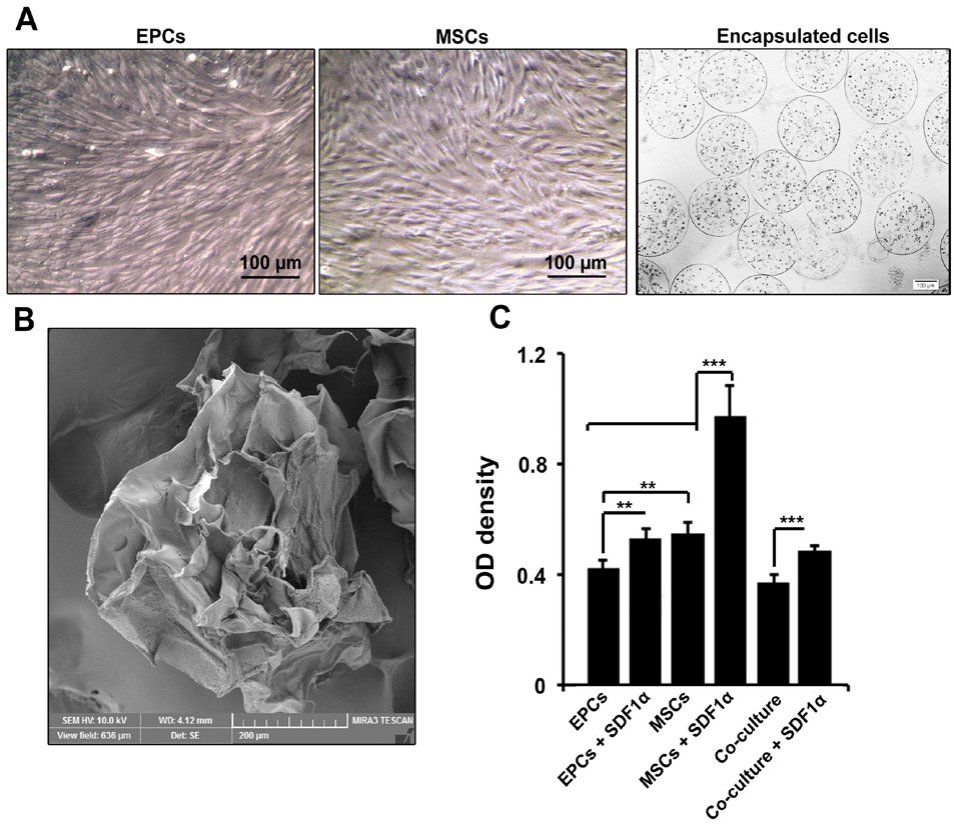
Microscopic bright field images of EPCs and MSCs in the plastic surface and inside alginate-gelatin microspheres (**A**). Both MSCs and EPCs acquired fibroblast-like appearance on the plastic surface while they were rounded-shape inside microspheres. Scanning electron microscopy of alginate-gelatin microspheres showed the existence of pores in the membrane shell (**B**). The data showed increased viability of MSCs in the presence of SDF-1α compared to other groups (**C**). These values were decreased in microspheres containing both types of EPCs and MSCs. One-way ANOVA and Tukey post-hoc analysis. **P  < 0.01 and ***P  < 0.001 (n=3).

According to data from bright field microscopic imaging, both EPCs, MSCs showed spindle-shaped morphology at passage three (Figure 1A). EPCs, MSCs encapsulation alone or in combination with each other acquired rounded shape inside alginate-gelatin microspheres (Figure 1A). Based on data, cells were evenly distributed inside microspheres, showing an appropriate cell encapsulation. SEM imaging showed a mean diameter of 430 ± 50.8 µm in alginate-gelatin microspheres (Figure 1B).

### Cell viability rate was regulated after EPCs and MSCs co-culture inside alginate-gelatin

MTT assay showed the enrichment of alginate-gelatin microspheres with SDF-1α promoted EPCs survival compared to control EPCs (*P*  < 0.01; Figure 1B). We also found that the encapsulation of MSCs with alginate-gelatin microspheres harboring SDF-1α increased cell viability compared to the control-matched MSCs and EPCs (*P*  < 0.001; Figure 1B). It seems that the conjugation of membrane shell with SDF-1α increased cell viability either in microcapsules containing EPCs or MSCs. However, these effects were prominent in MSCs (*P*  < 0.001; Figure 1B). Of note, the co-culture of EPCs and MSCs caused a decrease in cell viability compared to single cell encapsulation (*P*  < 0.001;Figure 1B). These data showed that alginate-gelatin encapsulation has different effects on cell viability related to distinct cell type.

### Co-culture of MSCs and EPCs inside alginate-gelatin promoted cell multipotentiality

**Figure 2:**
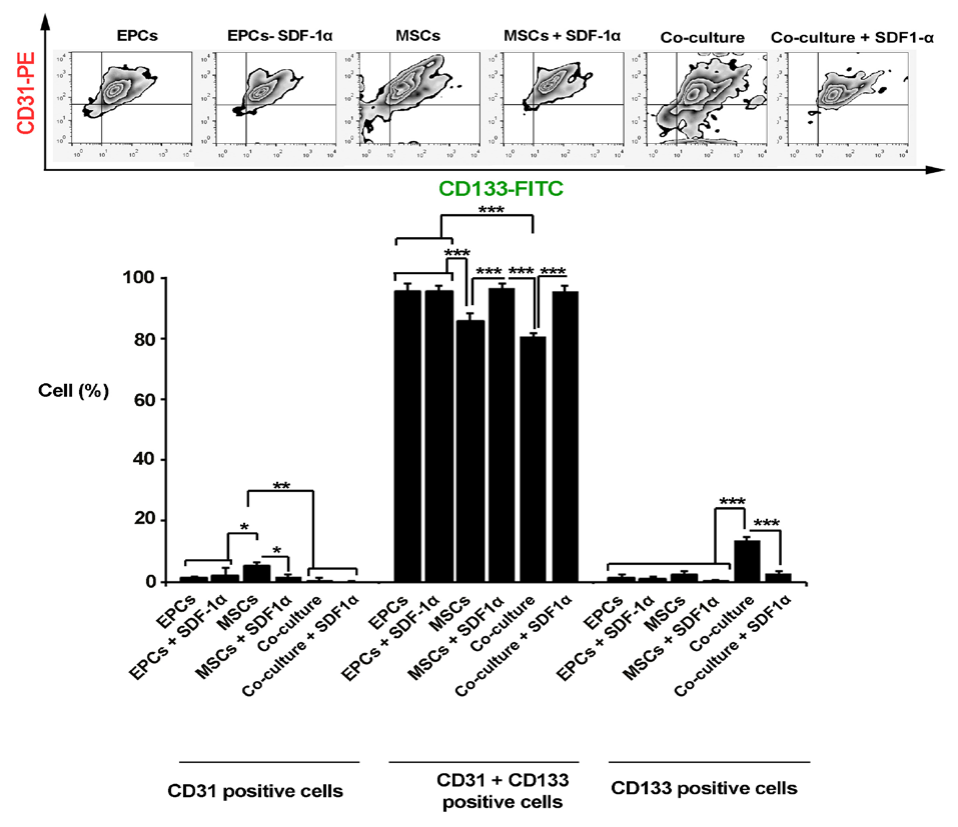
Flow cytometry analysis of encapsulated cells inside alginate-gelatin microspheres after 7 days. The data showed a significant increase in the percent of CD31 positive cells in MSCs groups. The simultaneous encapsulation of EPCs + MSCs caused an increase in the number of CD133 cells and stemness feature maintenance in the co-culture system compared to other groups. The addition of SDF-1α factor to a mixture of MSCs and EPCs caused a decrease of CD133 cells. One-Way ANOVA and Tukey post-hoc analysis. *P  < 0.05; **P  < 0.01 and ***P  < 0.001 (n=3).

Based on the data from flow cytometry panel, we found a significant increase in the number of CD133 positive cells and preservation of stemness feature when EPCs and MSCs were simultaneously co-cultured inside alginate gelatin microbeads compared to the matched control groups (*P*  < 0.001; Figure 2). The addition of SDF-1α factor to a mixture of MSCs and EPCs caused a decrease of CD133 cells. In single cultured groups either in MSCs or EPCs groups, the conjugation of SDF-1α to alginate gelatin membrane shell did not show significant differences compared to the control groups (*P* >0.05). Based on the analysis, the percent of CD31-positive cells were increased significantly in the MSCs group, showing the potential of alginate-gelatin in the induction of endothelial-like lineage compared to the EPCs (*P*  < 0.05). The addition of SDF-1α inhibited the endothelial differentiation of MSCs after 7 days (*P*  < 0.05; Figure 2). It seems that the EPCs maintained the multipotentiality inside the alginate-gelatin microspheres while MSCs showed superiority to trans-differentiated into the endothelial lineage.

### Simultaneous EPCs and MSCs co-culture inside alginate-gelatin promoted tubulogenesis

**Figure 3:**
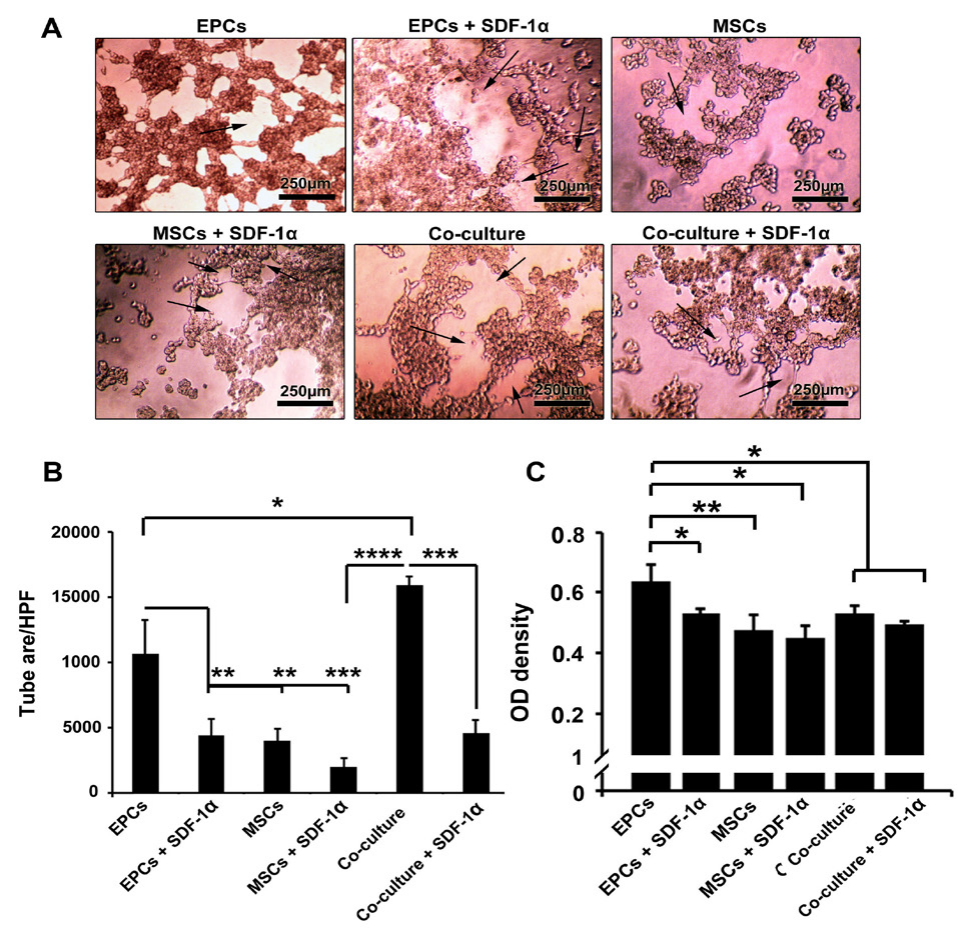
*In vitro* Matrigel tube formation assay post-encapsulation with alginate-gelatin microbeads (**A**-**B**). Based on data, the mean tube area was increased significantly in the presence of MSCs and EPCs. Compared to control EPCs, the addition of SDF-1α inhibited the tubulogenesis and vascular network formation of EPCs compared to SDF-1α-free condition (**A**-**B**). Detection of VE-cadherin protein by ELISA (**C**). The incubation of EPCs inside alginate-gelatin microbeads promoted the synthesis of VE-cadherin after 7 days. The VE-cadherin in control EPCs reached maximum level compared to MSCs and co-culture groups (**C**). One-way ANOVA and Tukey post hoc analysis. *P  < 0.05; **P  < 0.01 and ***P  < 0.001; *P*  < 0.0001 (n=3).

*In vitro* tubulogenesis assay showed that EPCs maintained the tubulogenesis capacity 7 days after encapsulation with alginate-gelatin microbeads (Figure 3A and B). The addition of SDF-1α inhibited the angiogenic potential and vascular network formation of EPCs compared to SDF-1α-free condition (*P*  < 0.01). We found a minimum tubulogenesis activity in microbeads harboring MSCs with or without SDF-1α compared to EPCs group (*P*  < 0.01). It seems that incubation of MSCs with EPCs inside alginate-gelatin microsphere increased tube formation activity in comparison with single encapsulated culture system (*P*_Co-culture vs. EPCs_ <0.05; *P*_Co-culture VS. EPCs + SDF-1α, MSCs_ <0.0001). The exposure of MSCs + EPCs with SDF-1α decreased tube formation capacity compared to SDF-1α-free co-culture condition (*P*  < 0.001;Figure 3A and B)

### SDF-1α loaded alginate-gelatin microspheres decreased VE-cadherin 

Results showed the incubation of EPCs inside alginate-gelatin microbeads promote the production of VE-cadherin after 7 days (Figure 3C). These levels were higher compared to MSCs groups (*P*  < 0.01). Based on our data, the enrichment of microbeads with 10 ng/mL SDF-1α decreased the EPCs ability to synthesize VE-cadherin compared to the matched control groups (*P*  < 0.05). As expected, the levels of VE-cadherin were lower in the co-culture system compared to the EPCs group (*P*  < 0.05). Non-significant results were observed in the co-culture system with or without SDF-1α. These data demonstrated that SDF-1α decreased cell-to-cell connection by decreasing the level of VE-cadherin.

### AC-Dil-LDL uptake capacity of cells was improved after SDF-1α treatment

**Figure 4:**
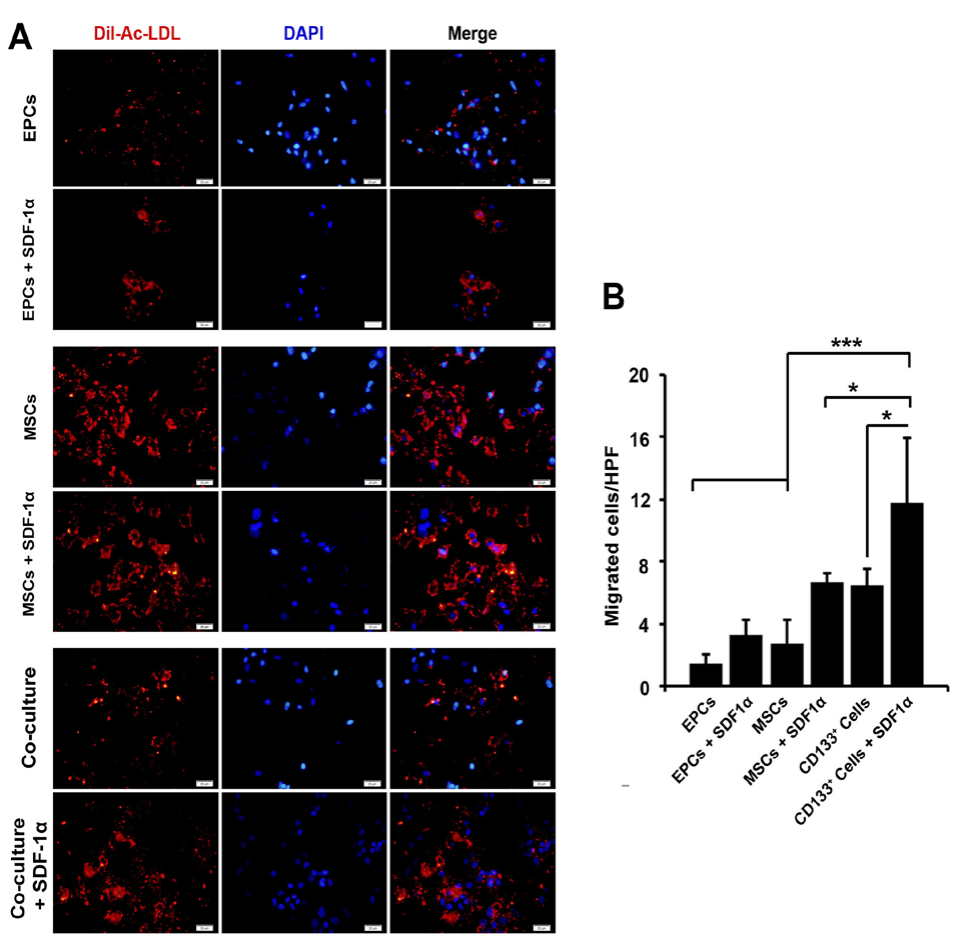
Immunofluorescence images of AC-DIL-ADL uptake activity in encapsulated cells with alginate-gelatin microbeads (**A**). Data showed that EPCs had a minimum level of intracellular AC-DIL-ADL, the addition of SDF-1α increased lipoprotein lipase activity. We found that encapsulated MSCs had the most AC-DIL-ADL uptake activity in the presence of SDF-1α (**A**). Measuring cell migration by using Transwell insert assay (**B**). The addition of SDF-1α had a significant effect on cell migration in the single and co-culture systems (4B). One-way ANOVA and Tukey post hoc analysis. *P  < 0.05; **P  < 0.01 and ***P  < 0.001 (*n* =3).

AC-Dil-LDL uptake capacity is touted as a functional capacity of endothelial lineage. Based on the data from the current experiment, we found that both EPCs and MSCs were able to uptake a large amount of AC-Dil-LDL seven days after encapsulation with alginate-gelatin microspheres compared to the parallel control-matched group (Figure 4A). In our experiment, these effects were more evident in EPCs compared to the EPCs in the condition with SDF-1α. In the co-culture system, the ability of cells to uptake AC-Dil-LDL was lower than the MSCs. These data show that the treatment of encapsulated cells with SDF-1α could promote endothelial-like functionality inside alginate-gelatin microspheres after 7 days.

### Cell migration was increased in the presence of SDF-1α

We noted that the addition of SDF-1α in EPCs, MSCs, and co-culture system inside alginate-gelatin microspheres induced the migration of cells 7 days after microencapsulation (Figure 4B). It seems that these effects were more prominent in the co-culture system compared to groups containing MSCs and EPCs (*P*  < 0.001; Figure 4B). The juxtaposition of EPCs and MSCs inside alginate-gelatin microspheres increased cell migration capacity compared to compared to SDF-1α-free condition (*P*  < 0.05; Figure 4B). Therefore, the application of SDF-1α seems to be an appropriate strategy in the induction of migration and motility.

### AKT and PK1 were efficiently up-regulated in encapsulated EPCs 

**Figure 5:**
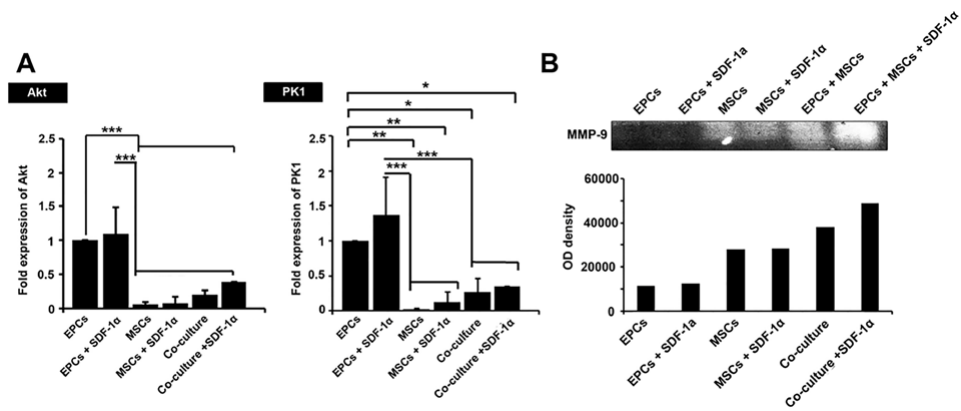
The real-time PCR analysis of genes *AKT and PK1* genes in encapsulated cells after 7 days (**A**). Data showed that the transcription level of all genes was up-regulated after the addition of SDF-1α. EPCs had had the most bioactivity to express *AKT* and*PK1* compared to MSCs and co-culture system (**A**). Gelatin zymography assay (**B**). The cells showed the most gelatinase activity (MMP-9 activation) when encapsulated with SDF-1α-loaded alginate-gelatin microspheres. In group EPCs + MSCs, we found the most gelatinase activity in the presence of SDF-1α compared to the other groups. One-Way ANOVA and Tukey post hoc analysis. *P  < 0.05; **P  < 0.01 and ***P  < 0.001 (n=3).

*AKT* and *PK1* participate in cell migration and multipotentiality. Based on the data from real-time PCR analysis, the addition of SDF-1α increased the expression of these genes in single and co-culture systems but did not reach significant levels compared to the control-matched groups (*P* >0.05; Figure 5A). Based on our data, EPCs had more ability to express *AKT* and *PK1* genes compared to the other groups (*P*  < 0.5; Figure 5A). It seems that the combined culture of EPCs and MSCs decreased the potency of EPCs to express genes *AKT* and *PK1* (*P* >0.05). We found the least ability to express *AKT* and *PK1* genes in MSCs either in SDF-1α-loaded or SDF-1α-free alginate-gelatin microspheres (Figure 5A).

### SDF-1α increased the degradation capacity of EPCs and MSCs 

Based on the data from gelatinase assay, all culture system except EPCs groups acquired potency to release MMP-9 and degrade gelatin after encapsulation with alginate-gelatin microspheres (Figure 5B). The gelatinase activity of MSCs either in the single or co-culture system was more than that of EPCs group. We found the highest degradation activity on the co-culture system. These data note that cell-to-cell connection from a different source, EPCs, and MSCs, could possibly promote gelatinase activity inside alginate-gelatin microspheres.

## Discussion

One of the challenges in the field of regeneration medicine is a low therapeutic efficiency after direct injection of distinct cell type into the target sites.^9^ In many circumstances, a decreased bioactivity, apoptotic changes, and a state of dormancy were seen in transplanted cells.^10^ It seems that microencapsulation of various cell types has several advantages by creating a 3D environment with an appropriate hydrodynamic phase inside aqueous systems. Indeed, the storage of cells behind a semi-permeable membrane provides a niche for cells to release growth factors while being away from immune cells.^11^ Angiogenesis is an integral procedure for successful regeneration and restoration of injured tissues after cell transplantation. This procedure is defined as the formation of nascent blood vessels and vascular collateralization.^12^ To encapsulate cells, different biomaterials alginate, collagen, fibrin, hyaluronic acid, and gelatin have been investigated extensively in various experiments.^13^ In most of the studies, alginate has been used as a backbone to support another substrate by providing a cross-link connection. However, due to an excessive anionic charge, the attachment of the cells seems to be decreased.^14^ Commensurate with these comments, the addition of natural extracellular matrix could circumvent the limitations and pitfalls correlated with alginate. Considering the ability of gelatin to form a niche for cell attachment and appropriate interaction with anionic polysaccharides, such as alginates, we used the mixture of alginate-gelatin for the fabrication of microspheres *in vitro.*^2^

To our knowledge, there are a few numbers of experiments related to the angiogenic potential of MSCs and EPCs co-culture inside alginate-gelatin microspheres. It seems that simultaneous encapsulation of two types of MSCs and EPCs could be an interesting area of study in the field of angiogenesis. According to data, we found a maximum level of cell viability in microspheres harboring MSCs and loaded with SDF-1α.^15^ It was stated that the addition of SDF-1α could increase the cellular viability in progenitor cells via engaging SDF-1α/CXCR-4 and -7.^16,17^ The co-culture of EPC-MSC inside alginate-gelatin initiated cell-to-cell contact soon after encapsulation that could be participated in the inhibition of cell viability compared to the single culture system.^18^ Previous data also demonstrated the diverse effects of SDF-1α dependent on cell type, dose and time manner.^19^

We also found that encapsulation of MSCs juxtaposed with EPCs yield a maximum level of marker CD31 in MSCs group while the level of VE-cadherin was more evident in the control EPCs. The addition of SDF-1α seems to maintain and increase the percent of CD133 and decrease the synthesis of VE-cadherin and CD31. However, the analysis of *PK1* (chemotaxis) and *AKT* (stemness) levels showed that EPCs showed a higher gene expression capacity compared to other groups. According to our data, SDF-1α could up-regulate these genes but the differences did not reach significant levels compared to parallel control. Consistent with these data, we showed magnificent tube formation capacity in microsphere harboring both cell types (EPCs and MSCs). In line with these statements, the addition of SDF-1α increased the migration of encapsulated cells. It seems that the use of SDF-1α not only could increase the cell multipotentiality, hampered angiogenic differentiation of both stem cell types, but also decreased cell-to-cell physical connection. Consistently, simultaneous encapsulation of EPCs and MSCs increased cells tubulogenesis. VE-cadherin is a cell membrane-associated protein that plays an important role in extracellular and EC-EC connections.^20^ In general, angiogenesis requires extracellular connections such as VE-cadherin and reduced EPCs migration rate.^21^ The decrease of VE-cadherin in a hydrogel with SDF-1α could diminish vascular network formation in a favor of stemness feature and cellular migration. In support of this claim, we found that in groups treated with SDF-1α the MMP-9 activity was increased which could participate in cells with migration activity. We further examined the uptake of LDL to address lipoprotein lipase activity of encapsulated cells. We found a high rate of LDL uptake capacity in MSCs and co-culture system. In a study conducted by Wang and colleagues, they showed that encapsulation of human embryonic stem cells inside alginate microspheres accelerated cell differentiation toward insulin-producing cells.^22^ Consistent with the hypothesis in this study, it has been shown that the application of SDF-1α in scaffolds could increase MSCs recruitment to the target site and decrease the cell dosage required for regeneration.^23,24^ These data showed that enrichment of backbone scaffold could improve regenerative potential of different stem cell types in favor of angiogenesis and vascular development.

There are some limitations related to the current experiment. To mimic the *in vivo* condition, we did not examine the effect of collagen and other ECM substrates. It seems that combination of different substrates could reflect the angiogenic potential of MSCs and EPCs inside alginate microspheres in a 3D condition.

## Conclusion

In conclusion, we found that the juxtaposition of EPC with MSCs increased in the Alginate-gelatin microspheres and induced differentiation of cells into endothelial cells and tubulogenesis. Moreover, SDF-1α had the potential to increase in AC-LDL uptake in MSCs and EPCs/MSCs. SDF-1α upregulated PK1 and Akt in encapsulated cells, especially in a co-culture system

## Ethical Issues

This study was approved by the ethical committee of Tabriz University of Medical Sciences (Ethical Code: 58906). All phases were in accordance with the principles of the Declaration of Helsinki.

## Conflict of Interest

All authors declare no competing financial interests exist

## Acknowledgments

The authors wish to thank the personnel of Stem Cell Research Center and Faculty of Advanced Medical Sciences, Tabriz University of Medical Sciences. This study was supported by a grant (no: 58906) from Tabriz University of Medical Sciences.
